# Relevance of the Pharmacokinetic and Pharmacodynamic Profiles of *Puerariae* *lobatae* Radix to Aggregation of Multi-Component Molecules in Aqueous Decoctions

**DOI:** 10.3390/molecules21070845

**Published:** 2016-06-28

**Authors:** Bili Su, Yongjun Kan, Jianwei Xie, Juan Hu, Wensheng Pang

**Affiliations:** 1 The Institute of Drug Research, Fujian Academy of Traditional Chinese Medicine, Fuzhou 350003, China; hzf_ketizu@163.com (B.S.); cld_ketizu@163.com (Y.K.); 2 The College of Pharmacy, Fujian University of Traditional Chinese Medicine, Fuzhou 350122, China; 13860662716@163.com

**Keywords:** *Puerariae lobatae* Radix decoction, molecular aggregation, pharmacokinetic profiles, pharmacodynamic profiles

## Abstract

The complexity of traditional Chinese medicines (TCMs) is related to their multi-component system. TCM aqueous decoction is a common clinical oral formulation. Between molecules in solution, there exist intermolecular strong interactions to form chemical bonds or weak non-bonding interactions such as hydrogen bonds and Van der Waals forces, which hold molecules together to form “molecular aggregates”. Taking the TCM *Puerariae* *lobatae Radix* (Gegen) as an example, we explored four Gegen decoctions of different concentration of 0.019, 0.038, 0.075, and 0.30 g/mL, named G-1, G-2, G-3, and G-4. In order of molecular aggregate size (diameter) the four kinds of solution were ranked G-1 < G-2 < G-3 < G-4 by Flow Cell 200S IPAC image analysis. A rabbit vertebrobasilar artery insufficiency (VBI) model was set up and they were given Gegen decoction (GGD) at a clinical dosage of 0.82 g/kg (achieved by adjusting the gastric perfusion volume depending on the concentration). The HPLC fingerprint of rabbit plasma showed that the chemical component absorption into blood in order of peak area values was G-1 < G-2 > G-3 > G-4. Puerarin and daidzin are the major constituents of Gegen, and the pharmacokinetics of G-1 and G-2 puerarin conformed with the two compartment open model, while for G-3 and G-4, they conformed to a one compartment open model. For all four GGDs the pharmacokinetics of daidzin complied with a one compartment open model. FQ-PCR assays of rabbits’ vertebrobasilar arterial tissue were performed to determine the pharmacodynamic profiles of the four GGDs. GGD markedly lowered the level of AT_1_R mRNA, while the AT_2_R mRNA level was increased significantly vs. the VBI model, and G-2 was the most effective. In theory the dosage was equal to the blood drug concentration and should be consistent; however, the formation of molecular aggregates affects drug absorption and metabolism, and therefore influences drugs’ effects. Our data provided references for the rational use of Chinese medicines in the clinic, such as the best oral preparation and decoction concentration.

## 1. Introduction

Traditional Chinese medicine is a great treasure trove of practical clinical knowledge accumulated over thousands of years and an important contributor to global health care. However, the complexity of traditional Chinese medicines (TCMs) is related to their multi-component systems, and their bizarre variety continues to present many unsolved enigmas [1]. The aqueous decoction of TCM is a common clinical dosage form that is prepared by blending every herb together then boiling them in water. There can be hundreds of chemicals in any given TCM decoction.

Molecules are held together by chemical bonds or weak non-bonding interactions such as hydrogen bonds and Van der Waals forces to form aggregates in a very general phenomenon. Drug aggregates might be absorbed through the Tran cellular pathway, epithelial cells that line the intestinal wall or via specialized microvillus cella (M cella) that cover the lymphoid follicles [2,3]. It had known that highly fat-soluble substances such vitamin A and vitamin D are transported by the lymphatic system and their absorption is good [4,5]. The gastrointestinal system is extensively permeated by a lymphatic network and the small intestine has the largest surface area for drug absorption in the GI tract, where drugs move from the oral to the systemic circulation [6]. This is the mechanism of drug intestinal absorption for highly lipophilic and poorly water-soluble compounds.

Previous works from the authors’ research group showed that molecular aggregate particles are ubiquitous in TCM water decoctions [7]. The efficacy of Chinese herbal formulas containing molecular aggregates against multiple targets was also correlated. TCM aggregation is beneficial for multiple components to form “molecular clusters” that act on the same target to explain the efficacy of the substance [8], but the oral bioavailability of drugs is negatively affected by the molecular scale; aggregation may compromise the stability as well as the biological activity of drugs.

Molecular size and shape can be determined in three ways. For example, the morphology of particles can be visualized using a scanning electron microscope (SEM); however, this method has one important limitation in that it only concerns a static target [9]. The particle size can be measured by dynamic light scattering (DLS), which is generally used as a batch technique to measure the average size in the whole sample, however, there is a lack of simultaneous detection of the particle shape.

Flow imaging microscopy uses a charge-coupled device camera with high magnification to capture images of a liquid sample passing through a thin flow cell. When the flow cell is illuminated, particles have a different refractive index, and the light intensity is lower in the solution compared to the background. Particle size and count information are generated based on analysis of the captured images. The digital particle images can supply morphological characterization data including shape, size, and optical parameters. However, this requires sufficiently high image quality to arrive at reliable conclusions. There are four flow imaging microscopy instruments available for biopharmaceuticals on the market [10]. Malvern Instruments offers several types of leading instruments for all types of particle size analysis and characterization from sub-nanometer to millimeters in particle size. Each system has its own advantages and disadvantages in different aspects. In recent years a wide range of instruments for wet and dry applications in particle size analysis and shape characterization has been made available by Occhio Instruments S.A. (Brussels, Belgium), using a unique optical design paying particular attention to high quality image acquisition, the efficiency of particle dispersion and orientation, and the accuracy of the particle measurements, which use robust algorithms [11].

*Pueraria lobata* (Willd.) Ohwi Root (Yege) is known as “Gegen”. It is used together with other complementary ingredients in brain nourishing formulas, active neck & shoulder care, and tension relief formulas. The chemical constituent puerarin, 3′-hydroxypuerarin, 3′-methoxypuerarin and daidzin dilate blood vessels, increase coronary blood flow, have anti-thrombotic effects, inhibit platelet aggregation, reduce blood viscosity and promote blood micro-cycling. Pueraria has good effects on cervical spondylosis. Angiotensin II receptor, type 1 or AT1 receptor is the best characterized angiotensin receptor. It is an important effect or controlling blood pressure and volume in the cardiovascular system. The octapeptide angiotensin II (Ang II) plays a homeostatic role in the regulation of blood pressure and water and electrolyte balance, and contributes to the progression of blood vessel remodeling. Ang II activates Ang II type 1 receptor (AT1R) and type 2 receptor (AT2R), both of which belong to the seven-transmembrane, G protein-coupled receptor family. Most of the actions of Ang II such as promotion of cellular proliferation, hypertrophy, and fibrosis are mediated by AT1R. However, in some pathological situations, AT2R showed an increase in expression level and functions to antagonize the actions by AT1R stimulation. Like Fenge, Gegen contains too much starch, and starch facilitates the formation of molecular aggregates. Many factors affect molecular aggregate size, especially the concentration of the solution [12]. So far, however, no study has investigated the permeation behavior of different concentrations of Gegen aqueous decoction (GGD).

**Figure 1:**
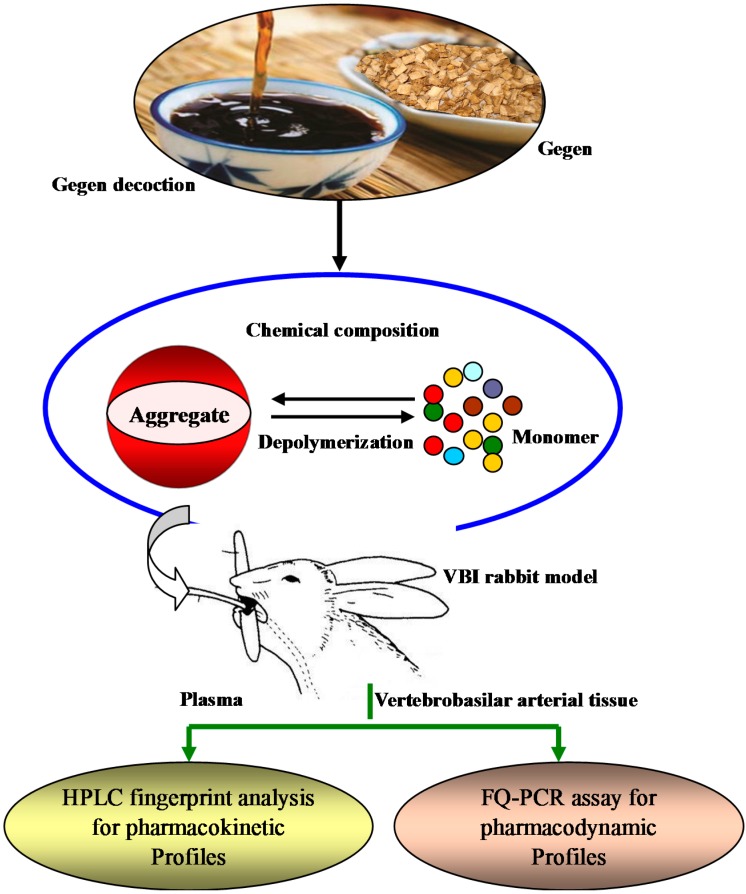
Diagram of the focus of this paper.

In this paper, we take GGD as an example, and a new image-processing system (Flow Cell, Occhio S.A.) was tested, which allows the simultaneous analysis of size and shape of the aggregation morphology of particles in GGD [13]. Thus, the aim was to provide information for certain concentration(s) of GGD exhibiting oral bioavailability. Rabbits with a vertebrobasilar artery insufficiency (VBI) model were given different concentrations of GGD at a clinical dosage of 0.82 g/kg (obtained by adjusting the gastric perfusion volume depending on concentration). High performance liquid chromatography (HPLC) analysis was used as a tool to examine how GGD with different molecular oligomers influenced the pharmacokinetic profiles. Using the fluorescence quantitive polymerase chain reaction (FQ-PCR) method the mRNA expressions of angiotensin II type 1 receptor (AT_1_R) and angiotensin II type 2 receptor (AT_2_R) of vertebrobasilar arterial tissue of the VBI model were detected to obtain evidence of the relation between the molecular aggregate sizes of GGD and their pharmacodynamic profiles (Figure 1). This study may be beneficial for correlation with quality control in the Chinese medicine preparation industry if the size and shape of mainly large or irregularly formed particle clusters are of interest.

## 2. Results and Discussion

### 2.1. Particle Size and Shape in the Four GGDs

**Figure 2:**
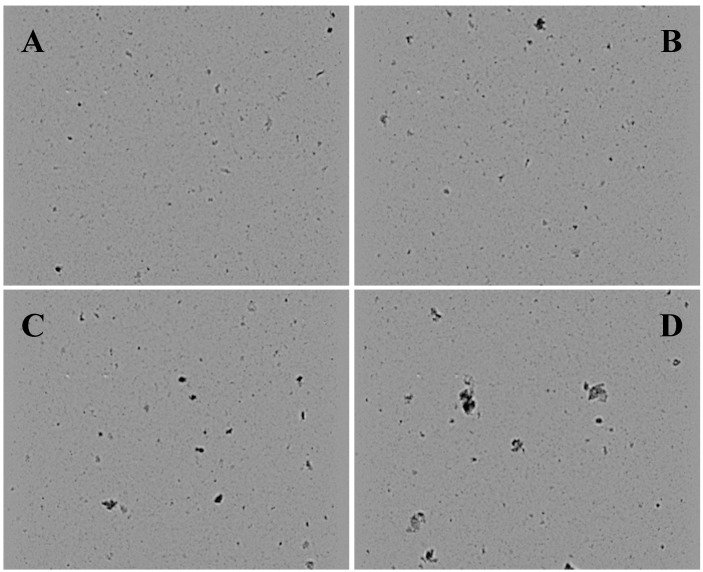
Images taken by the Flow-Cell: (**A**) G-1 solution; (**B**) G-2 solution; (**C**) G-3 solution; (**D**) G-4 solution; scale bar 100 µm.

Four decoctions of different concentration (0.019, 0.038, 0.075, 0.30 g/mL, respectively) were named G-1, G-2, G-3, G-4 and used to study the effect of different molecular aggregates on the particle size and shape. Visualization with the Flow Cell of G-1 (Figure 2A) showed particles with an average inner diameter from 10.14 µm up to 28.10 µm. G-2 (Figure 2B) showed particles of 10.80 µm in average inner diameter up to 30.10 µm. G-3 (Figure 2C) showed particles with 22.42 µm with an average inner diameter up to 31.96 µm. G-4 particles were molecular clusters; the smaller ones represent small molecular aggregates, while the larger ones represent the interaction of smaller molecules—protein or starch (Figure 2D); particles were 36.20 µm in average inner diameter up to 50.49 µm. Thus, some particles might not have been detected with the Flow Cell due to the chosen pixel size (here: 0.38 µm) meaning that the pixel size is a limiting parameter. In contrast, the G-1 and G-2 solutions (Figure 2A,B) revealed mainly approximately ball shaped granules. G-3 solution (Figure 2C) revealed elongated particles with various lengths. In the G-4 solution (D) larger particles were visible with irregular, mostly convex shapes.

**Figure 3:**
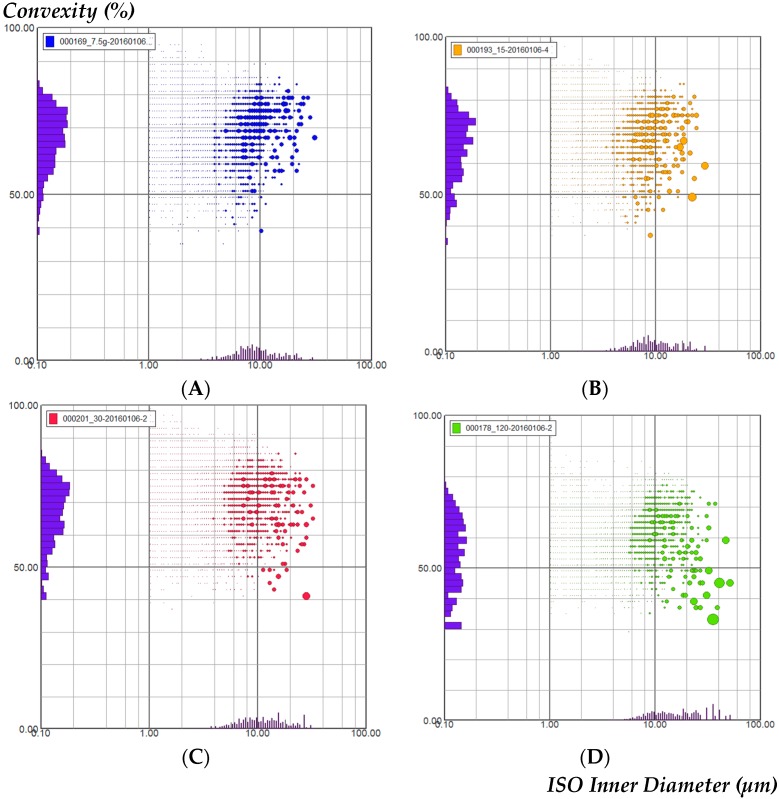
Analysis of the particle convexity and size of (**A**) G-1 decoction; (**B**) G-2 decoction; (**C**) G-3 decoction; (**D**) G-4 decoction.

To analyze the particle shapes in the G-1, G-2, G-3, and G-4 solutions; the convexity parameter (distribution-related and shape-related) was chosen as a good differentiation was expected (Figure 3). The ratio of the data for each parameter is given on the axis. In G-1 solution (Figure 3A) most particles had an ISO inner diameter between 6–40 µm and a distribution of 0.40–1.0, suggesting mainly convex rather than concave particle parts; G-2 solution (Figure 3B) showed an ISO inner diameter (6–40 µm) distribution (0.4–1.0); there was no obvious difference between the convexity of G-1 and G-2. However, G-3 solution (Figure 3C) showed an ISO inner diameter (6–40 µm) distribution (0.45–1.0) and G-4 solution (Figure 3D) showed an ISO inner diameter (8–80 µm) distribution (0.5–1.0), the latter indicating a more concave shape. The result showed that the concentration of GGD was significantly correlated with the degree of molecular aggregation.

The aggregate size increased as the concentration of GGD increased. The order of the four kinds of solution in order of molecular aggregate size (diameter) was G-1 < G-2 < G-3 < G-4. The complexity of traditional Chinese medicines (TCMs) is related to their multi-component systems. TCM aqueous decoction is a common clinical oral formulation. Between molecules in solution, there exist strong intermolecular interactions to form chemical bonds or weak non-bonding interactions such as hydrogen bonds and Van der Waals forces, whereby molecules are held together to form “molecular aggregates”.

### 2.2. HPLC Fingerprints of Rabbit Plasma

The frozen rabbit plasma was thawed at room temperature. Plasma samples (600 µL) were precipitated with methanol in 1:4 proportion. After vortex mixing for 5 min, the specimens were centrifuged at 10,000 rpm/min for 10 min. The supernatant was pipetted off and dried very quickly under N_2_. The solid matter as vortexed and dissolved in about 150 µL of methanol. After filtration (0.45 μm micro-porous filtering film) plasma samples (20 µL injection volume) were analyzed by HPLC.

**Figure 4:**
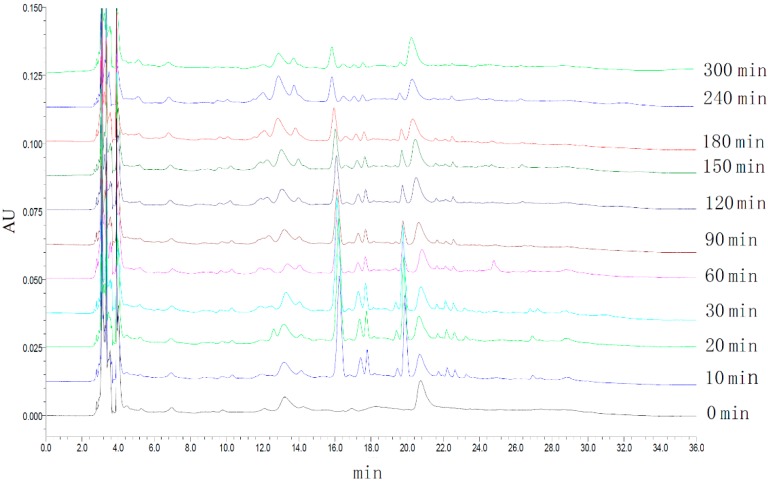
The HPLC fingerprint of rabbit plasma of G-2 decoction at different times.

The HPLC fingerprints of rabbit plasma were determined at different times. The average of peak area (*n* = 3) was used as the characteristic variable. GGD was absorbed and removed quickly, and the drug concentration reached a maximum in 30 min; rabbits can metabolize various ingredients of GGD in 300 min (see Figure 4).

**Figure 5:**
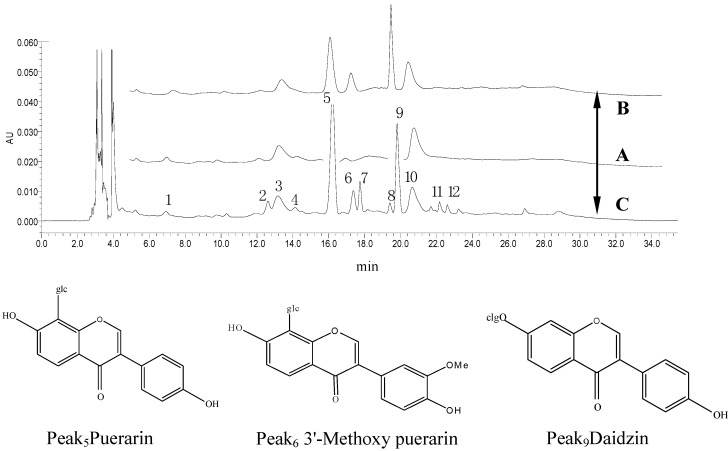
The HPLC chromatogram of Gegen decoction: (**A**) Blank rabbit plasma sample; (**B**) blank rabbit plasma samples piked with puerarin (10 µg/mL), 3′-methoxypuerarin (3.6 µg/mL) and daidzin (7 µg/mL); (**C**) rabbit plasma sample at 35 min after their stomachs were perfused with 0.82 mg/kg G-2 solution. The chromatographic peaks were identical between standards and sample. The three structures are shown above.

Under the experimental HPLC conditions, the rabbit plasma fingerprints showed that there were nine exogenous substance absorbed into blood in G-2 solution (labeled as 2, 4–9, 11, 12), and three endogenous substances (labeled as 1, 3, 10). Peaks 5 and 9 were the major constituents absorbed into blood. The chromatographic comparison between standards and samples of the three peaks 5, 6 and 9 showed they were identical to puerarin, 3′-methoxypuerarin and daidzin (Figure 5). Chemical components were absorbed into blood in the order of peak area value G-1 < G-2 > G-3 > G-4.

### 2.3. Rabbit Plasma HPLC Analysis of the Pharmacokinetic Profiles of the Indicative Components Puerarin and Daidzin in GGD

#### 2.3.1. Specificity and Linearity

The rabbit plasma method specificity was evaluated by comparing blank plasma after oral administration of GGD at 0.82 g/kg. No endogenous substance or other interfering peaks were observed at the retention time of puerarin and daidzin, indicating that the method was specific.

The calibration curve method was adapted to measure the plasma concentrations of puerarin and daidzin. The regression equation of peak area (*y*) to tested concentration (*x*) was Puerarin: *y* = 220160*x* + 3015.5 with a linear range from 0.051–5.1 mg/L; Daidzin: *y =* 232185*x* + 16505 with a linear range from 0.035–3.5 mg/L; The calibration curve showed good linearity over the plasma concentration with correlation coefficients (*r*) of 0.9998 and 0.9991, respectively.

#### 2.3.2. Accuracy and Precision

The method presented satisfactory repeatability and reproducibility, with intraday (*n* = 5) and interday (*n* = 5, 3 days) precision of less than 3.59% and accuracy ranging from 1.66% to 3.09% for the analysis of the three compounds. These results confirmed that the method was sensitive enough for the pharmacokinetic study of puerarin and daidzinin rabbits.

#### 2.3.3. Pharmacokinetic Profiles of Puerarin and Daidzin

**Figure 6:**
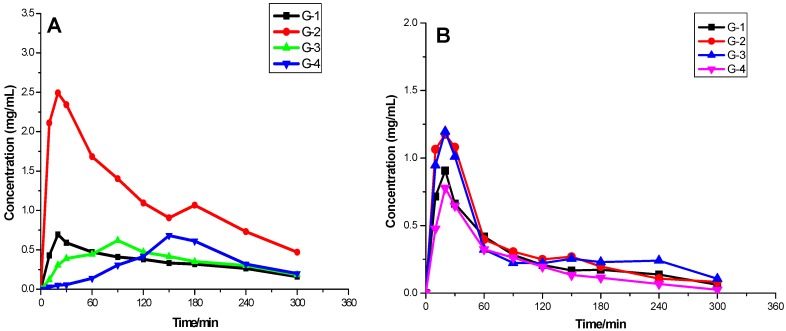
The plasma concentration-time curves of puerarin and daidzin after rabbit stomachs were perfused with four kind of GGD with dosages equal of 0.82 g/kg (*n* = 4, $\bar{x}\pm sd$). (**A**) The plasma concentration-time curves of puerarin; (**B**) mean plasma concentration-time curves of daidzin.

After rabbit stomachs were perfused with Gegen decoction of dosage equal of 0.82 g/kg, using four kinds of GGD with different aggregation at different time points for comparative analysis (Figure 6) the concentration of puerarin and daidzin in rabbit plasma versus time curves revealed that the G-1, G-2 groups reached the maximum concentration (C_max_) of puerarin at about 20 min. For the G-3 and G-4 groups, the C_max_ of puerarin was detected at 90 min and 150 min, respectively. Comparing the different time points, in the G-2 group, the average puerarin concentration was higher than in the other three groups, showing that the G-2 group was more easily absorbed into the blood, and the bioavailability was high. With increasing size of the aggregates, the bioavailability decreased gradually. For daidzin, in the G-1, G-2, G-3, and G-4 groups, the C_max_ were all detected at 20 min. Comparing different time points, in the G-2 and G-3 groups, the drug blood concentrations were similar; G-2 and G-3 group differ from G-1 and G-2 in drug blood concentration, but the difference is small. It may be that the absorption, distribution, metabolism and excretion of daidzin were less affected by the state of the aggregates. The pharmacokinetic data was subjected to non-compartmental analysis using the DAS 2.0 pharmacokinetic analysis software. The main pharmacokinetic parameters of puerarin and daidzin were calculated by a non-compartment model analysis. The data are presented in Table 1 and Table 2.

After rabbit stomachs were perfused with GGD, puerarin and daidzin was rapidly absorbed into the blood. The half-life times for elimination (t_1/2α_) of G-1, and G-2 were 5.15 ± 2.01 min, 17.79 ± 9.82 min, respectively, whereas the half-life elimination (t_1/2β_) times of G-3, G-4 were 69.32 ± 3.58 min, 65.28 ± 4.93 min, respectively, indicating that puerarin was relatively quickly eliminated from plasma with a short elimination half-life in the G-1, G-2 groups. The maximum plasma drug concentrations (C_max_) of puerarin were 0.69 ± 0.16, 2.62 ± 0.61, 0.62 ± 0.05, 0.038 ± 0.063 mg/L corresponding to G-1, G-2, G-3, G-4; and the values of AUC_0–t_ were 106.9 ± 5.96, 357.9 ± 5.25, 110.5 ± 6.34, and 60.00 ± 10.88 mg/L min, respectively. There are statistically significant differences in the average values of V_1_/F, CL/F, T_max_, C_max_, AUC_0–t_, and AUC_0–∞_ of puerarin among the G-1, G-2, G-3, and G-4 groups. The AUC_0–t_ and values of G-2 reached the maximum, but V_1_/F and CL/F of G-2 showed minimal values. The pharmacokinetics in G-1 and G-2 of puerarin conformed to a two compartment open model and the oral absorption is rapid, while the elimination half life is short. In the G-3 and G-4 groups, the pharmacokinetics of puerarin conformed to a one compartment open model (Table 1).

The half-life elimination (t_1/2α_) times of daidzin were 56.29 ± 2.55, 25.29 ± 4.44, 10.56 ± 2.44, 8.02 ± 0.80 min, respectively; there were no difference of t_1/2β_ which were all about 69 min (*p* > 0.05). The maximum plasma drug concentrations (C_max_) of daidzin were 0.91 ± 0.10, 1.24 ± 0.38, 1.12 ± 0.24, 0.78 ± 0.14 mg/L for G-1, G-2, G-3 and G-4. The values of AUC_0–t_ were 82.79 ± 7.33, 104.8 ± 3.05, 102.9 ± 2.66, and 65.54 ± 3.71 mg/L min, respectively. Except for the V_1_/F and t_1/2α_ values, there are no statistically significant differences in the average CL/F, T_max_, and C_max_ values of daidzin among the G-1, G-2, G-3, G-4 groups. The values of AUC_0–t_, and AUC_0–∞_ were similar for both the G-2 and G-3 groups and were larger than for the G-1 and G-4 groups. In all four groups the pharmacokinetics of daidzin conformed to a one compartment open model (Table 2).

### 2.4. Pharmacodynamic Profiles of the Four GGDs in the Anti-Rabbit Vertebrobasilar Artery Ischemia Model

#### 2.4.1. Transcranial Doppler to Evaluate the Vertebrobasilar Artery Ischemia Model of Rabbits

**Figure 7:**
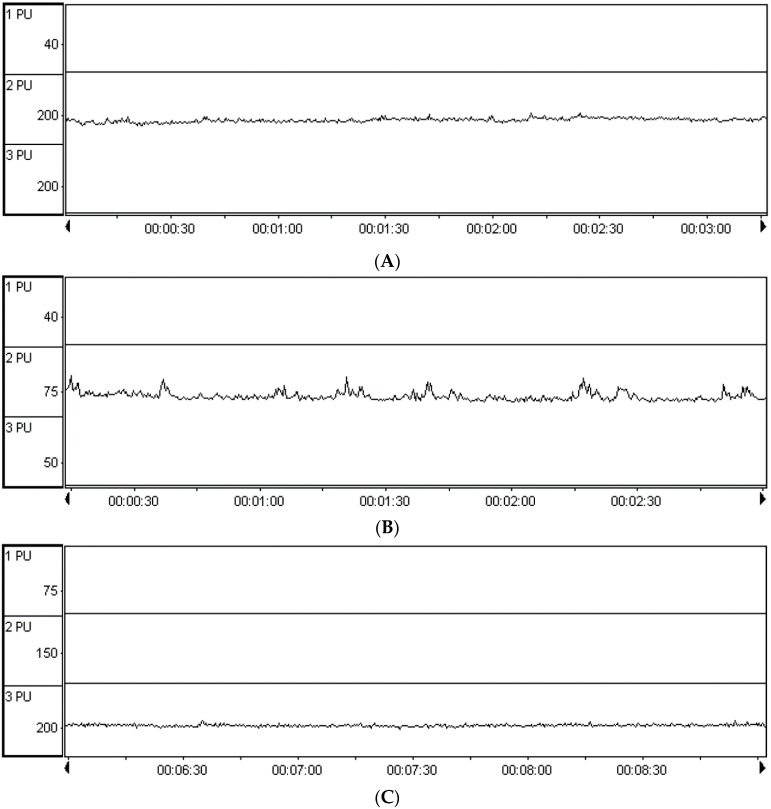

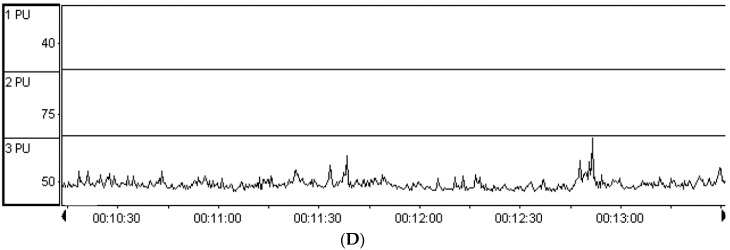
The diagram for blood flow (Vm) determined by means of a Transcranial Doppler (TCD) instrument. (**A**) Blank control and (**B**) VBI model in the vertebral artery; (**C**) blank control and (**D**) VBI model in the basilar artery.

Rabbits were injected with tissue sclerosing agent 775 injections in the left side of the cervical vertebrae to induce an animal model of chronic vertebroarterial artery ischemia. One month later, the hair of the occipitoposterior area and neck were shaved off and the animals were sedated with intravenous injection of a dose of 200 mg/kg of 10% chloral hydrate to tranquilize them. The probe was aimed at the cervical vertebra side and external occipital protuberance posterior occipital window to detect the blood flow (Vm) of the vertebral artery (VA) and basilar artery (BA) by means of a Transcranial Doppler (TCD) instrument. The vertebral artery and base artery perfusion provide direct evidence of vertebrobasilar artery ischemia. The value (PU) Vm in the blank control group was 21.84 ± 0.036 dm/s; Vm decreased to 5.60 ± 0.24 dm/s in VA after modeling; the value (PU) of Vm in the blank control group was 16.02 ± 0.091 dm/s; Vm decreased to 6.99 ± 0.081 in BA after modeling. In the VA or BA, there are thus statistically significant differences in the average values of Vm between blank control and model group (*p* < 0.01). The vertebrobasilar artery ischemia model was thus proved to be successfully implemented (Figure 7). The results of hemodynamic changes after the four GGD treatments, Vm and BA values all increase in VA animal; see Table 3.

#### 2.4.2. Rabbits Vertebrobasilar Arterial Tissue FQ-PCR Assay for the Pharmacodynamic Profiles of the Four GGDs

Purified PCR product can also be used to measure pharmacodynamic profiles; quantification by OD_260_ and OD_280_ is influenced by any products in the sample. The DNA purity was judged according to the ratio of OD_260_/OD_280_. For the high purity DNA the OD_260_/OD_280_ value is in the range of 1.7–2.0, and values below this range indicate that the protein content is impermissibly high, while values higher than this range indicate that the sample contains RNA. Using ethidium bromide (EB) as a fluorescent probe, agarose gel electrophoresis showed three clear bands of 18S rRNA, 5S rRNA and 28S rRNA. The experimental OD_260_/OD_280_ ratio of rabbit basal artery plasmids in each group was between 1.8 and 2.0 and OD_260_/OD_230_ was more than 2, which showed that the DNA purity was high.

**Figure 8:**
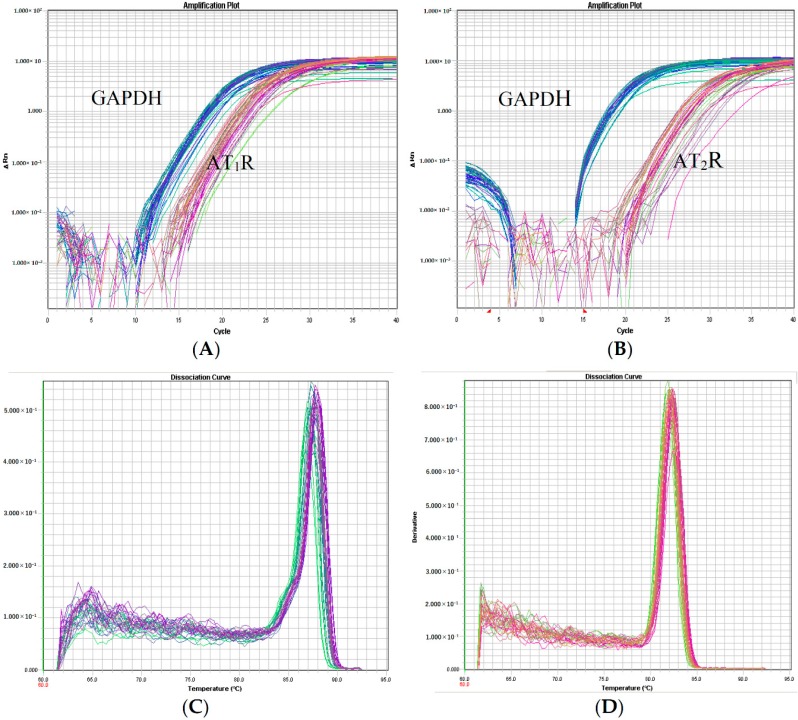
The amplification plots and dissociation curves of AT_1_R and AT_2_R: (**A**,**B**) amplification plots of AT_1_R and AT_2_R; (**C**,**D**) dissociation curves of AT_1_R and AT_2_R.

During the PCR amplification, the DNA polymerase activity cleaves the target-specific hybridization probe and releases the reporter dye. The increase in fluorescence emission was proportional to the amount of PCR product accumulated, which in turn was proportional to the starting target concentration. Fluorescence emission was monitored, in real time, using a sequence detector. Figure 8 shows amplification plots of two-fold serial dilutions of total RNA from rabbits’ vertebrobasilar arterial tissue. Amplification of the AT1R and AT2R were observed from the fifteenth or twentieth cycles onwards, as indicated by an increase in the fluorescence intensity. AT1R, AT2R, GAPDH dissociation curve of the three genes were unimodal, proving that the amplified products were from specific genes (Figure 8).

The gene expression of AT1R mRNA and AT2R mRNA was tested in rabbits’ vertebrobasilar arterial tissue with relative quantization by the ^△△^Ct Method. The AT1R mRNA gene level was 1.02 ± 0.070 (blank control group) which increased to 2.81 ± 0.20 after VBI modeling; 2.42 ± 0.25 for the G-1 group, 1.29 ± 0.33 for the G-2 group, 1.51 ± 0.26 for the G-3 group, and 2.21 ± 0.34 for the G-4 group. After using G-2 and G-3 the gene expression recovery of AT1R mRNA was remarkable (*p* < 0.01), while that using G-1 and G-4 was not remarkable (*p* > 0.05).

The gene level of AT2R mRNA in the blank control group was 1.02 ± 0.033, which decreased to 0.29 ± 0.061after VBI modeling; in the G-1 group it was 0.49 ± 0.12, 1.06 ± 0.17 in the G-2 group, 0.99 ± 0.14 in the G-3 group and 0.31 ± 0.022 in the G-4 group. After using G-2 the gene expression recovery of AT2R mRNA was remarkable (*p* < 0.01), while using G-3 it was greatly improved (*p* < 0.05), and using G-1 and G-4 it was not remarkable (*p* > 0.05, Table 3).

## 3. Experimental Section

### 3.1. Materials

Gegen medicinal slices meeting Chinese Pharmacopoeia regulations (2015) produced by Shanghai Kangqiao Chinese Medicine Tablet Co., Ltd., China (Shanghai, China) was purchased from Fujian Xiangan Drug Limited Company (Quanzhou City, China). Manufactured products undergo strict quality testing procedures, and are proven to be qualified for delivery in conformity with the standards. HPLC grade methanol and acetonitrile were bought in Merck (Darmstadt, Germany). Analytical grade reagents were bought in Sinopharm Chemical Reagent Co. (Shanghai, China). Hanks’ balanced salts (HBSS) and resazurin were purchased from Sigma–Aldrich (Shanghai, China). Water was deionized by a Milli-Q-Plus ultra-pure water system (Millipore, Milford, MA, USA).

### 3.2. Methods

#### 3.2.1. Samples Preparation

Dried Gegen medicinal slices were ground to 60 mesh powder and 7.50, 15.0, 30.0 and 120.0 g weighed out. Six times cold-water was added to soak them for 30 min, followed by boiling for 15 min and simmering 15 min, decocting two times. The decoction was strained through a piece of gauze and the two filtrates combined into one. The volume was made up to 400 mL, and the concentrations of the four decoctions, which were named G-1, G-2, G-3, G-4, respectively, were 0.019, 0.038, 0.075, 0.30 g/mL.

#### 3.2.2. Characterization of Molecular Aggregation

The particle size and shape were measured with the Flow Cell 200S IPAC image analyzer (Occhio S.A.). A certain sample volume is pumped through the measuring cell and irradiated with monochromatic, collimated (parallel aligned) light (λ = 440 nm). Images are taken with a high resolution camera (6.6 Megapixel) fitted with at elecentric lens, processed, and analyzed with the Windows-compatible programme Call is to. In general, particle sizes from 0.1 to 2500 μm are measurable depending on the focus adjustment and the mounted spacer (in our case: 400 μm spacer, pixel size 0.38 μm). A syringe diameter of 2060 μm, cell thickness 100 μm, analysis quantity 0.05 mL was used. The Flow Cell calculates the particle size distribution based on the size of each detected particle.

#### 3.2.3. Rabbit Plasma HPLC Analysis for Pharmacokinetic Profiles of the Four GGDs

After fasting 24 h the first time rabbits were given GGD at doses 0.82 g/kg by gastric perfusion. Blood (3 mL) was taken from the ear middle artery of each rabbit at different time points (0, 10, 20, 30, 60, 90, 120, 150, 180, 240, 300 min), and placed in a polystyrene tube containing heparin sodium. The sample was centrifuged 10 min at 3000 rpm and 4 °C and the supernatant was immediately frozen for storage at −20 °C.

HPLC analysis was done using an Alliance 2695 separation module system (Waters Technologies, Milford, MA, USA), equipped with an auto-sampler, a quaternion pump, mobile phase degasser, temperature-controlled auto sampler, column thermostat, photodiode array detectors (2998 PAD). A Hypersil C_18_ column (4.6 mm × 250 mm, 5 μm) from Dalian Elite Analytical Instruments Co., Ltd. (Dalian, China) was used. The chromatograms were analyzed by the Empower Waters Chromatography Data System Version 3.0. Acetonitrile and 0.2% formic acid aqueous solution were used as eluent A and eluent B. Detection wavelength was set to 250 nm with a flow of 1.0 mL/min and an injection volume of 20 µL. Column temperature was set to 30 °C. A gradient elution program was applied; run time was at 30 min with eluent A from 8% up to 30%, then down to 15%, while eluent B was down from 92% to 70%, then up to 85% (Table 4).

#### 3.2.4. Animal Model Preparation and VBI Rabbit Treatments

Twenty four New Zealand rabbits evenly divided between males and females weighing between 2.5–3.0 kg were fed food and water ad libitum. The VBI model group rabbits were injected with 200 g/L tissue sclerosing-775 injection (10 mL) into the soft tissue on the left side of the C_2–6_ lateral transverse process of the cervical vertebrae, and injected once again on the second week [14]. The control group was injected with an equal volume of saline. The dynamic vertebrobasilar blood flow was monitor with a Transcranial Doppler (TCD) device to evaluate the VBI model at the 4th week [15].

Drug groups and VBI model rabbits were treated with four kinds of different concentration of Gegen decoction administrated at the same dose of 0.82 g/kg. Normal control and model group animals received a single oral dose of the same volume of saline; all groups were treated for four weeks. This study was approved by the Fujian University of Traditional Chinese Medicine Ethics Committee.

#### 3.2.5. Rabbits Vertebrobasilar Arterial Tissue FQ-PCR Assay for Pharmacodynamic Profiles of the Four GGDs

The rabbits were sedated with a 200 mg/kg intravenous dose of 10% chloral hydrate before undergoing surgery and the vertebrobasilar arterial tissue were collected. DNA extraction was performed using a 74,104 RNeasy^®^ Mini Kit (Qiagen, Shanghai, China) following the manufacturer’s instructions, and then the extracted RNA was stored at −80 °C. A FQ-PCR assay on 7500 Fast Real-Time PCR System (Applied Bio-Systems, Waltham, MA, USA) was used to test the effects on gene expression of AT_1_R, AT_2_R of vertebrobasilar arterial tissue in VBI model rabbits fed with the four GGDs, respectively.

The DNA gene sequences of AT_1_R, AT_2_R and internal reference GAPDH were obtained from the Genebank website and then the PCR primers of AT1R and AT2R were designed. The sequences for the two pairs of primers were: AT1R: F 5′-TGTGTCTTATAGGTTTACACTGC-3′, R 5′-TTCATACTCATTCAAGGTAGTCT-3′; AT2R: 5′-TGGCTCTTTGGACCTGTGATGTG-3′, R 5′-CGGAAATAAAATGTTGGCAATG-3′; GAPDH: F 5′-CAACGGGAAACCCATCACCA-3′, R 5′-ACGCCAGTAGACTCCACGACAT-3′. The 50 µL reaction system contained 10 µL reaction buffer, 10 µL Taq Mix, 2 µL cDNA, and 1 µL upstream primer, down stream primer and AT1R, AT2R, and GAPDH probe, respectively. Five µL extracted DNA was supplemented with distilled water (dH_2_O) to 50 µL. An Applied Bio-systems 7500 Fast PCR system was employed for the amplification reaction, with the reaction conditions as follows: 50 °C for 2 min, 95 °C for 10 min, 55 °C for 15 s, with 40 repeats, 60 °C for 30 s, with 40 repeats. The fluorescence signal was recorded at 60 °C.

### 3.3. Statistical Analysis

All data was examined with SPSS 15.0 for statistical analysis. Basic data are analyzed by means of *t*-test or ANOVA a done-way ANOVA for comparing more than two independent means. All values are expressed as mean ± SD, while *p* < 0.05 indicated a significant difference.

## 4. Conclusions

Rabbits were given different concentration of four GGDs (G-1, G-2, G-3, and G-4) at clinical dosages of 0.82 g·kg^−1^ (the gastric perfusion volume was adjusted according to the concentration). In theory the dosage was equal, so the blood drug concentration should be consistent, but the HPLC fingerprint results indicated that there was a marked difference in plasma substances among the four groups. Puerarin and daidzin are the major constituents of Gegen, and there was a marked difference between the pharmacokinetics of the two compounds governed by the concentration of the Gegen decoction. Many interactions are involved in *Radix puerariae* that can form aggregates, such as hydrogen bonds and Van der Waals forces. In many cases, knowing just the sample concentration is not enough, as particle size may also affect drug absorption, distribution, metabolism, or the biological response to drug delivery that is far from being completely understood. Our data provides references for the rational use of this Chinese medicine in the clinic, such as the best oral preparation and decoction concentration.

## Figures and Tables

**Table 1:** Pharmacokinetic parameters of puerarin after rabbit stomachs were perfused with four kinds of GGD of dosage equal to 0.82 g/kg (*n* = 4, $\bar{x}\pm sd$)^R^.

Parameter	Unit	G-1	G-2	G-3	G-4
t_1/2_	min			69.32 ± 3.58	65.28 ± 4.93
t_1/2α_	min	5.15 ± 2.01 ^▼▼^	17.79 ± 9.82		
t_1/2β_	min	69.32 ± 1.82	69.32 ± 2.88		
V_1_/F	L/kg	446.1 ± 8.53 **^,▼▼^	192.9 ± 9.22 **	527.4 ± 7.23 **^,▼▼^	975.1 ± 10.28 **^,▼▼^
CL/F	L/min/kg	8.38 ± 6.38 **^,▼▼^	1.74 ± 0.19 **	5.27 ± 0.74 **^,▼▼^	10.46 ± 1.96 **^,▼▼^
T_max_	min	21.32 ± 1.35 **^,▼▼^	26.45 ± 5.77 **	91.12 ± 3.80 **^,▼▼^	157.5 ± 1.67 **^,▼▼^
C_max_	mg/L	0.69 ± 0.16 **^,▼▼^	2.62 ± 0.61 **	0.62 ± 0.05 **^,▼▼^	0.038 ± 0.063 **^,▼▼^
AUC_0–t_	mg/L·min	106.9 ± 5.96 **^,▼▼^	357.9 ± 5.25 **	110.5 ± 6.34 **^,▼▼^	60.00 ± 10.88 **^,▼▼^
AUC_0–∞_	mg/L·min	147.5 ± 9.85 **^,▼▼^	474.6 ± 7.58 **	158.1 ± 2.65 **^,▼▼^	80.34 ± 1.11 **^,▼▼^

Remarks: *t*-Test (Two Populations); G-1, G-3, G-4 vs. G-2, ^▼▼^*p* < 0.01; Comparisons among four groups were tested by one-way ANOVA analysis ** *p* < 0.01; no significant differences between groups—no label.

**Table 2:** Pharmacokinetic parameters of daidzin after rabbit stomachs were perfused with four kinds of GGD of dosage equal to 0.82 g/kg (*n* = 4, $\bar{x}\pm sd$)^R^.

Parameter	Unit	G-1	G-2	G-3	G-4
t_1/2α_	min	56.29 ± 2.55 **	25.29 ± 4.44 **	10.56 ± 2.44 **	8.02 ± 0.80 **
t_1/2β_	min	69.31 ± 2.35	68.62 ± 1.20	69.31 ± 1.82	69.31 ± 3.12
V_1_/F	L/kg	676.4 ± 15.63 **^,▼▼^	220.8 ± 7.98 **	238.5 ± 8.71 **	369.3 ± 6.22 **^,▼▼^
CL/F	L/min/kg	7.81 ± 1.55	6.93 ± 1.60	5.72 ± 2.99	11.32 ± 0.54 ^▼^
T_max_	min	20.00 ± 4.08	22.50 ± 2.30	22.50 ± 3.22	20.00 ± 2.06
C_max_	min	0.91 ± 0.10	1.24 ± 0.38	1.12 ± 0.24	0.78 ± 0.14 ^▼^
AUC_0–t_	mg/L·min	82.79 ± 7.33 ^▼^	104.8 ± 3.05	102.9 ± 2.66	65.54 ± 3.71 ^▼^
AUC_0–∞_	mg/L·min	108.1 ± 3.99 ^▼^	122.7 ± 8.40	115.82 ± 7.58	72.54 ± 3.45 ^▼▼^

Remarks: *t*-Test (Two Populations); G-1, G-3, G-4 vs. G-2, ^▼▼^*p* < 0.01, ^▼^*p* < 0.05. Comparisons among four groups were tested by one-way ANOVA analysis ** *p* < 0.01; no significant differences between groups—no label.

**Table 3:** Different concentrations of GGDs’ effect on blood flow data and AT_1_R mRNA/AT_2_R mRNA expression ($\bar{x}\pm sd$, *n* = 4).

Groups	Vm (dm/s)	BA (dm/s)	2^−^^△△^^ct^(AT_1_R)	2^−^^△△^^ct^(AT_2_R)
Blank control	21.84 ± 0.036	16.02 ± 1.47	1.02 ± 0.070	1.02 ± 0.033
Model control	5.60 ± 0.24 **	6.99 ± 0.081 **	2.81 ± 0.20 **	0.29 ± 0.061 **
G-1	6.92 ± 1.33	7.73 ± 0.32	2.42 ± 0.24	0.49 ± 0.12 *
G-2	18.38 ± 0.79 ^▲▲^	14.16 ± 1.47 ^▲▲^	1.29 ± 0.33 ^▲▲^	1.06 ± 0.17 ^▲▲^
G-3	17.29 ± 1.36 ^▲▲^	15.63 ± 0.95 ^▲▲^	1.51 ± 0.26 ^▲▲^	0.99 ± 0.14 ^▲▲^
G-4	10.14 ± 1.28 ^▲^	10.42 ± 1.56 ^▲^	2.21 ± 0.34	0.31 ± 0.022

Model control vs. control group: ** *p* < 0.01, * *p* < 0.05; GGD group vs. model group: ^▲▲^ *p* < 0.01, ^▲^ *p* < 0.05.

**Table 4:** HPLC method conditions for the analyses.

*t*/min	Solvent/%	
	Acetonitrile	0.2% Formic Acid
0	8	92
13	15	85
14	20	80
16	20	80
18	25	75
25	30	70
30	15	85
